# Nutritional Calorie Labeling and Menu Ordering Practices Among US Adults With Chronic Illnesses

**DOI:** 10.7759/cureus.58484

**Published:** 2024-04-17

**Authors:** Chukwuka Ibecheozor, Justin Morales, Jillian Ross, Adaeze Ezeofor, Charmaine McKie, Victor F Scott, Angesom Kibreab, Charles Howell, Farshad Aduli, Hassan Brim, Hassan Ashktorab, Mosunmola Oyawusi, Shelly McDonald-Pinkett, Adeyinka O Laiyemo

**Affiliations:** 1 Department of Medicine, Johns Hopkins Hospital, Baltimore, USA; 2 Department of Psychiatry, New York University, New York, USA; 3 Department of Medicine, Georgetown University, Washington, D.C., USA; 4 Department of Medicine, Massachusetts General Hospital, Boston, USA; 5 Department of Clinical Research, Mid-Atlantic Permanente Research Institute, Mid-Atlantic Permanente Medical Group Largo Medical Center, Largo, USA; 6 Department of Medicine, Howard University College of Medicine, Washington, D.C., USA; 7 Department of Pathology, Howard University College of Medicine, Washington, D.C., USA; 8 Department of Neurology, Howard University College of Medicine, Washington, D.C., USA

**Keywords:** dietary fiber, obesity, nutrition and metabolism, comorbid obesity, hypertension, diabetes mellitus, calorie intake, chronic disease epidemiology, food labeling

## Abstract

Background: The relationship between diet and the management of chronic illnesses is well established. However, it is unknown the extent to which people with chronic illnesses pay attention to nutritional information and act upon the information obtained. We evaluated the menu ordering practices of adults with chronic illnesses.

Methods: We analyzed the 2018 Health Information National Trends Survey (HINTS 5 Cycle 2). Our analytic cohort included 3,154 respondents (weighted population size=228,464,822) who answered questions regarding a personal history of diabetes, hypertension, heart disease, and obesity. They also answered questions about their nutritional habits regarding whether they noticed caloric information at fast-food or sit-down restaurants and how that information influenced their dietary choices.

Results: Among respondents with these chronic illnesses, only obese patients were significantly more likely to pay attention to caloric information (OR=1.56; 95%CI: 1.06-2.31). However, noticing the calorie information was not associated with ordering less calories among all categories of respondents with chronic illnesses.

Conclusion: US adults with chronic illnesses do not pay sufficient attention to the calorie information of their diet. Furthermore, awareness of the calorie information did not influence their dietary choices. Healthcare professionals should incorporate dietary counseling into the management of chronic illnesses of their patients.

## Introduction

Numerous studies have established the connection between diet and the progression of chronic illnesses such as cardiovascular diseases including hypertension and diabetes [1-3] including the need for healthy dietary habits to prevent and manage these illnesses [4,5]. Our diet plays a critical role in the management of chronic illnesses as it is a modifiable risk factor for many chronic conditions including hypertension, diabetes, and heart disease [6,7]. Many epidemiological studies, randomized prevention trials, and short-term studies evaluating outcome measures such as blood pressure and lipids have revealed significant connections between specific dietary and lifestyle modifications and the management of major chronic illnesses [8-10]. These findings are important, because they indicate that these chronic conditions, in some cases, can be managed without drugs or expensive medical facilities [7].

Nutrition labeling is currently one of the primary methods used to achieve the goal of food consciousness. In the 1990s, the United States started using nutrition labels in an attempt to modify food intake by giving ingredient contents and nutrition values, and it is now mandatory for all processed foods [11,12]. It has been predicted that the requirement of nutrition labeling by the US Food and Drug Administration (FDA) could improve the health of the population by preventing cardiovascular diseases and cancer with a substantial reduction in healthcare costs in 20 years [13]. The FDA updated the labeling requirements to include sugar, vitamin D, and potassium while emphasizing that "calories" should be prominently displayed [14]. Dietary interventions such as the Dietary Approach to Stop Hypertension (DASH) diet and Mediterranean diet as well as food guides such as Choose My Plate and the Healthy Eating Plate are part of the efforts being made to guide dietary habits and prevent/manage chronic illnesses. Nutrition labeling is expected to enhance the adoption and adherence to dietary recommendations.

Strategies for managing chronic conditions are multifactorial, where dietary interventions and lifestyle modifications are typically regarded as first line. A general conclusion is that reducing modifiable dietary and lifestyle risk factors could prevent most cases of coronary artery disease, stroke, diabetes, and many cancers. Therefore, knowledge of dietary decisions and their impact is imperative when assessing the effectiveness of nutritional interventions in chronic conditions. As more data becomes available and new dietary plans are created, it remains uncertain whether adults with chronic illnesses use them and modify their dietary practices based on the information posted on the food [6,7]. In this study, we evaluated the menu ordering practices of adults with chronic illnesses in terms of whether they paid attention to the calorie information provided and if they modified their menu based on the information obtained.

An abstract from this study was presented as a poster at the Virtual Scientific Meeting of the American College of Gastroenterology in October 2020.

## Materials and methods

The Health Information National Trends Survey (HINTS) is a nationally representative survey conducted by the National Cancer Institute (NCI). This survey asks questions about health-related information and behavior of US adults. The HINTS collects data about the knowledge of, attitude towards, and use of health-related information among Americans. The 2018 HINTS 5 Cycle 2 data were collected from January to May 2018. The survey was conducted exclusively via mail, and toll-free phone numbers were provided to respondents to voice questions, concerns, or request materials in Spanish. More information about the HINTS 5 Cycle 2 survey can be found on the official website of the NCI at https://hints.cancer.gov/ [15].

After obtaining exempt approval from the Institutional Review Board of Howard University in Washington, D.C. (IRB-14-MED-28), we downloaded the dataset. Our analytic cohort included 3,154 respondents (weighted population size=228,464,822) who answered questions regarding a personal history of the following chronic illnesses: diabetes, hypertension, heart disease (including heart attacks, angina, or congestive heart failure), and obesity. We evaluated individuals with the presence of one of the chronic illnesses, and our outcome of interest was their response to questions about their nutritional habits regarding whether they noticed caloric information at fast-food or sit-down restaurants and how that information influenced their dietary choices. In the survey, participants were asked to respond "yes" or "no" to the following question: "Think about the last time you ordered food in a fast-food or sit-down restaurant. Did you notice calorie information listed next to the food on the menu or menu board?". Respondents who answered "yes" were asked follow-up questions including "Thinking about the last time you noticed calorie information on the menu or menu board, how did the calorie information change what you were thinking of ordering?". The respondents chose whether they ordered something with fewer calories. Individuals with and without particular chronic illness were compared. We evaluated whether they noticed caloric information on food labels and if they ordered less calories after being exposed to this information.

We used logistic regression analyses to examine the association between chronic illnesses and the likelihood of noticing caloric information along with modifying their dietary behavior by ordering less calories. We used survey weights in all analyses. We calculated odds ratios (OR) and 95% confidence interval (CI). Our final model included age, sex, smoking, income, race ethnicity, and highest education attained (Table 1).

**Table 1:** Characteristics of the survey respondents

Total population of the cohort	3154 (weighted population size=228,464,822)
Mean age in years	48.6 (95% CI: 47.9-49.4)
Sex, n (weighted %)	
Male	1271 (48.8%)
Female	1883 (51.2%)
Smoking, n (weighted %)	
Non-smoker	1935 (63.7%)
Former smoker	769 (20.5%)
Current smoker	417 (15.8%)
Income in dollars (weighted %)	
<20k	521 (16.7%)
20-34k	397 (11.6%)
35-49k	390 (13.8%)
50-74k	544 (18.0%)
75k+	1069 (39.9%)
Race ethnicity, n (weighted %)	
White	1866 (65.2%)
Black	404 (10.7%)
Hispanic	430 (15.9%)
Other	245 (8.2%)
Highest education attained, n (weighted %)	
High school or less	801 (30.4%)
Some college	943 (40.3%)
College graduate	1396 (29.3%)

## Results

A total of 3,154 respondents (weighted population size=228,464,822) to the 2018 HINTS 5 Cycle 2 were included in the study. The mean age was 48.6 years and 51.2% of the respondents were female and 15.8% were current cigarette smokers. Table 1 shows the characteristics of the respondents. In terms of noticing nutritional labels of foods (Table 2), only obese patients were more likely to notice caloric information on nutritional labels (OR=1.56; 95% CI: 1.06-2.31). Among all patients with chronic illnesses who noticed caloric information, there was no increased likelihood to act on the information obtained (Table 3). Having a chronic illness was not associated with ordering less calories among US adults.

**Table 2:** Association between chronic illnesses and likelihood of noticing caloric information Multivariate model adjusted for age, sex, smoking, income, race, and education

Chronic illness	Noticed calorie information	Univariate OR (95% CI)	Multivariate OR (95% CI)
Diabetes (n, weighted %)	n (weighted %)		
No (2,455, 84.0%)	1,110 (45.8%)	Reference	Reference
Yes (647, 16.0%)	279 (40.8%)	0.82 (0.62-1.08)	1.1 (0.82-1.46)
Hypertension (n, weighted%)			
No (1,704, 64.6%)	800 (47.3%)	Reference	Reference
Yes (1,401, 35.4%)	589 (41.0%)	0.77 (0.60-0.99)	0.98 (0.73-1.32)
Heart disease (n, weighted %)			
No (2,820, 93.5%)	1,295 (45.9%)	Reference	Reference
Yes (298, 6.5%)	100 (33.6%)	0.60 (0.41-0.87)	0.86 (0.61-1.23)
Obesity (n, weighted %)			
No (2,027, 65.2%)	880 (43.4%)	Reference	Reference
Yes (1,025, 34.8%)	488 (48.1%)	1.19 (0.68-1.38)	1.56 (1.06-2.31)

**Table 3:** Association between chronic illnesses and likelihood of ordering fewer calories Multivariate model adjusted for age, sex, smoking, income, race, and education

Respondents with chronic illness who noticed calorie information	Ordered fewer calories after noticing calorie information	Univariate OR (95% CI)	Multivariate OR (95% CI)
Diabetes (n, weighted %)	n (weighted %)		
No (1,110, 45.8%)	714 (59.7%)	Reference	Reference
Yes (279, 40.8%)	189 (68.2%)	1.45 (0.82-2.56)	1.36 (0.68-2.72)
Hypertension (n, weighted %)			
No (800, 47.3%)	543 (61.9%)	Reference	Reference
Yes (589, 41.0%)	358 (59.1%)	0.89 (0.59-1.36)	0.70 (0.48-1.03)
Heart disease (n, weighted %)			
No (1,295, 45.9%)	846 (60.8%)	Reference	Reference
Yes (100, 33.6%)	58 (63.0%)	1.01 (0.58-2.07)	1.00 (0.52-1.95)
Obesity (n, weighted %)			
No (880, 43.4%)	598 (64.5%)	Reference	Reference
Yes (488, 48.1%)	288 (54.4%)	0.75 (0.43-1.30)	0.81 (0.47-1.40)

## Discussion

In this study, we used the HINTS 5 Cycle 2 to evaluate if patients with chronic illnesses pay attention to nutritional labeling and how they are influenced by the information obtained. We found that, among all respondents with chronic illnesses, only obese patients were significantly more likely to pay attention to caloric information when compared with non-obese respondents. However, obese respondents did not alter their menu ordering practices by ordering fewer calories after noticing the calorie information. Similarly, among other respondents with chronic illnesses (diabetes, hypertension, and heart diseases) who noticed caloric information, the information did not influence their menu ordering practices also when compared to respondents without the specific chronic illness. Our finding was surprising as we had hypothesized that those with chronic illnesses were more likely to pay attention to nutritional labeling and modify their dietary choices based on this information. These results indicate that a lot more must be done by care providers and policymakers to improve the nutrition of patients with chronic illnesses in the United States.

In a similar study, Lewis et al. [16] reported that among 5,603 participants in the 2005-2006 US National Health and Nutrition Examination Survey (NHANES), patients with hypertension, hyperlipidemia, diabetes, obesity, and cardiovascular disease use nutrition labels more frequently than their healthy counterparts although this did not consistently translate into better dietary habits. In contrast, our study found no increased likelihood of utilizing nutritional labels among survey participants with chronic illnesses, except for obesity. However, both studies found that nutritional label use did not translate into better dietary habits even though the study by Lewis et al. was conducted more than a decade earlier. This showed that little or no progress has been made in this regard.

Our findings are also comparable to the report by Hong et al. [17]. In their study of 10,695 respondents to the 2008-2009 Korea National Health and Nutrition Examination Survey, the authors reported that respondents with chronic illnesses of hypertension, diabetes, and hyperlipidemia did not use nutritional labels differently from those without the same illnesses [17].

The intention of nutrition information and labeling is to promote healthy food choices and to guide food decision-making. In contrast, the present findings revealed that simply providing this information did not significantly influence US adults' dietary choices. Furthermore, a study conducted by Chen et al. [18] suggested that 80% of the United States use nutrition labels in some form but that socioeconomic status was a major determinant of nutrition label use and understanding. The authors also noted that overweight Americans with inaccurate self-perception of body weight are less likely to use food labels.

De la Cruz-Góngora et al. [19] reported that 17% of adult Mexicans use nutrition labels but only 1.2% could answer a five-question basic understanding test on nutrition labels. Similarly, Sharf et al. [20] also reported that only 27.2% of 120 young adults could properly understand the contents of nutrition labels. A major concern regarding nutritional label use is patients' understanding of dietary decisions and ability to interpret nutritional information adequately. Therefore, nutritional education should be personalized to the specific patient. This was suggested by Leyvraz et al. [21] who conducted a survey involving 588 participants aged 25-65 years in five sub-Saharan African countries, namely, Benin, Guinea, Kenya, Mozambique, and Seychelles. They concluded that a high intake of salt was a major risk factor in the development of hypertension and cardiovascular diseases and suggested that improving the knowledge, attitudes, and practices in relation to salt intake was a useful strategy in mitigating the impact of these chronic illnesses.

The key to changing dietary patterns is not only nutritional labeling but also patient education. Therefore, healthcare professionals should integrate nutritional and dietary counseling to help their patients [10,22]. This can be accomplished by increasing nutritional curriculum in medical education, emphasizing the translation of diet to clinical practice, which would enable healthcare professionals to serve as first-line providers of nutritional support and education; collaborating with nutritional experts such as dieticians more frequently; and tailoring nutritional needs to the specific patient profile [21].

Our study has some notable strengths which included the fact that we used a nationally representative data and studied a large number of US adults. However, our study has some limitations too. Our findings are based on self-reports. Our data analysis was also limited to the nature of the questions included in the survey which was related to meals prepared by others. Furthermore, we could not explore additional respondents' usage of nutritional labels such as the utilization of nutritional labels while grocery shopping.

## Conclusions

Although an established relationship exists between diet and the management of chronic illnesses, US adults with chronic illnesses do not utilize the nutritional information provided to them by public resources to improve their diet. Furthermore, awareness of caloric information did not influence their reported dietary choices. Healthcare professionals should stress the importance of diet in the long-term management of chronic illnesses of their patients and make appropriate referrals to nutritionists for optimal nutritional counseling.
